# Characteristics of Chronic Cough in adults in Pakistani population: A cross sectional study

**DOI:** 10.12669/pjms.36.3.1868

**Published:** 2020

**Authors:** Kaleem Ullah Toori, Asma Chaudhry

**Affiliations:** 1 Dr. Kaleem Ullah Toori, FRCP (Glasgow). Department of Medicine, KRL Hospital, Islamabad, Pakistan; 2 Dr. Asma Chaudhry, MRCP (UK). Department of Medicine, KRL Hospital, Islamabad, Pakistan

**Keywords:** Chronic cough, Causes, Asthma, Allergic rhinitis, ILD, ACE-I, GERD, BHR, Age, Gender, BMI

## Abstract

**Background and Objective:**

Data about causes of chronic cough are lacking in our part of world. The aim of our study was to look for spectrum of causes in our setup and to determine a correlation between causes of cough and baseline factors of age, gender, and BMI and compare it to other populations.

**Methods:**

Total 236 chronic cough patients who attended chest clinic at KRL Hospital Islamabad from January 2018 to June 2019 were included in this cross-sectional study. Chronic cough was defined as cough greater than eight weeks. Main causes of chronic cough taken were cough variant asthma, allergic rhinitis, interstitial lung disease, Gastro-esophageal reflux disease, bronchial hyper-reactivity, ACE-I induced cough and others’. Other demographic and clinical data were also recorded.

**Results:**

The mean age was 45.16± 16.50 years and BMI was 26.23 ± 4.68kg/m^2^. Major cause of chronic cough was cough variant asthma in 111(47%). Age had significant positive correlations with ILD, ACE-I induced cough and CCF, while significant negative correlations with CVA and AR. On gender correlation, ILD and ACE-I cough were significantly found more in females. BMI had significant correlation with ACE-I cough only.

**Conclusion:**

Variability of epidemiology of cough variant asthma, allergic rhinitis and ACE-I induced cough is comparable to worldwide data while differences exist with epidemiology of interstitial lung disease. Further research is needed in the field to delineate the local trends in this regard and compare to other population groups.

## INTRODUCTION

Chronic cough is broadly explained as a cough that lasts for at least eight weeks. Approximately 10 -20 percent of the population worldwide is affected by cough that is chronic in nature.^1^ The commonest causes include postnasal drip syndrome generally after viral infection of the upper respiratory tract, cough variant asthma and gastroesophageal reflux disease. Other significant causes include excessive vasomotor responsiveness, chronic sinusitis, chronic bronchitis or smokers, medications especially ACE-Inhibitors and irritants.^2^ Less common causes may include recurrent tonsillitis, parenchymal lung diseases, cancers and chronic infections.^2^

In patients with a normal chest radiograph, no definitive history of smoking or use of drugs especially angiotensin-converting enzyme (ACE) inhibitors, upper airway syndrome, cough variant asthma, and gastroesophageal reflux disease (GERD) are the most recognized causes of chronic cough.^1^,^2^

The causes of chronic cough differ depending on multiple factors including age distribution, gender, and weight. Causes of cough in younger age group include asthma, allergic rhinitis and sinusitis while in older age group include parenchymal lung diseases, medications and tumors. Similarly, when gender distribution is considered, some causes including asthma appear to be more common in females while others like lung cancer are more common in males.^3^

Data about the causes of cough are lacking in our part of the world. The aim of our study was to look for spectrum of causes in our setup and to determine if there was a correlation between the causes of cough and baseline factors of age, gender, and weight in our population.

## METHODS

It was a hospital-based descriptive cross-sectional study, carried out in KRL Hospital Islamabad where a consecutive series of patients with chronic persistent cough referred to our chest clinic between January 2018 and June 2019 were studied. The sample size calculated was 38, using OpenEpi Calculator with prevalence of chronic cough as 2.5%,^4^ margin of error as 5% with 95% confidence level, and we gathered data of 236 patients. The technique used for sampling was non-probability consecutive sampling. The purpose and benefits of the study were explained to all patients and a written informed consent was obtained. The study was conducted with approval from hospital ethical and research committee.

Individuals irrespective of gender, weight and age above or equal to eighteen years with chronic cough were included in the study. Demographic details and results of all patients were collected on a pre-designed Performa. All investigations were carried out according to routine procedure and standard methods of investigations at KRL Hospital.

Chronic cough was defined as cough persistent for more than or equal to eight weeks.^2^ BMI was calculated for all using standard height and weight machines. BMI groups were defined as underweight (below 18.5 kg/m^2^), normal (18.5-25 kg/m^2^), overweight (25-30 kg/m^2^), obese (30 to 40 kg /m2) and very obese (above 40 kg/m)^2^.^5^ Elaborative history was taken including history of smoking, diurnal variation of symptoms, gastroesophageal reflux, preceding respiratory tract infections, post-nasal drip, use of medication i.e. ACE-Inhibitors, family history of asthma, non-pulmonary co-morbidities like diabetes, hypertension, ischemic heart disease and chronic kidney or liver disease.

Chest x-ray and spirometry with reversibility were performed for all patients; results were recorded and reported by radiologist. Final diagnosis after laboratory work-up and imaging was recorded. Cough variant asthma was diagnosed with combination of history and spirometry results. Post nasal drip/Allergic rhinitis as diagnosed by symptomology and ENT evaluation, bronchial hyper-reactivity (BHR) by clinical history of chest infection followed by cough and subsequent improvement and High Resolution CT of chest was performed to ascertain interstitial lung disease (ILD). GERD was diagnosed by gastroenterologist following upper GI endoscopy, aided by history and examination. Tuberculosis was diagnosed by combination of history, chest x-ray and positive staining & cultures, lung carcinoma by CT chest with contrast and biopsy and Smoker’s cough in chronic smokers when other causes of cough were ruled out. Heart failure was diagnosed on echocardiography. Echocardiography was reported by cardiologist and CT scans were reported by radiologist.

Data was analyzed using IBM-SPSS version 22.0. Simple descriptive statistics were used for calculating frequencies, mean values and percentages. Mean and standard deviation were calculated for continuous variables like age and BMI. Frequency and percentages were calculated for categorical variables like gender and chronic cough causes. Research strategy was a correlational study. Spearman’s non-parametric correlation was used to analyze variations between causes of chronic cough and baseline factors of age, gender and BMI. P-value less than 0.05 was considered statistically significant.

## RESULTS

Total 236 patients were analyzed in this study. The mean age of patients was 45.16± 16.50 years, while mean BMI was 26.23 ± 4.68 kg/m^2^. There were 142 (60.2%) female patients and 94 (39.8%) male patients. Out of total dataset, 218 (92.4%) were non-smokers, while 18 (7.6%) were smokers. Patient characteristics are presented in Table-I.

**Table-I:** Patient Characteristics.

	Number of individuals N
Age (in years) - Mean ± SD	45.16± 16.50
*Age group (in years)- n (%)*	
<20	9 (3.8)
21-40	93(39.4)
41-60	88 (37.3)
>60	46 (19.5)
*Gender- n (%)*	
Male	94 (39.8)
Female	142 (60.2)
BMI(kg/m^2^) - Mean ± SD	26.23 ± 4.68
*BMI Groups- n (%)*	
Underweight	10 (4.2)
Normal	64 (27.1)
Overweight	81 (34.3)
Obese	38 (16.1)
Very Obese	2 (0.8)
Smokers – n (%)	18 (7.6)

The main causes of chronic cough taken in our study were cough variant asthma, allergic rhinitis, GERD, BHR, ILD and ACE-I induced cough. Other causes included tuberculosis, Smokers’ cough, lung malignancy and congestive cardiac failure (CCF). Frequencies of causes occurring in our patients are presented in Table-II.

**Table-II:** Frequency of causes of cough.

Causes of Cough	Number of individuals N
Cough variant asthma – n (%)	111 (47)
Allergic rhinitis – n (%)	29 (12.3)
GERD – n (%)	18 (7.6)
BHR – n (%)	18 (7.6)
ILD – n (%)	24 (10.2)
ACE-I induced cough- n (%)	14 (5.9)
Others (Total) - n (%)	15 (16.4)
Tuberculosis- n (%)	9 (3.8)
Lung Malignancy- n (%)	3 (1.3)
Smoker’s cough- n (%)	5 (2.1)
Heart failure- n (%)	5 (2.1)

**GERD -** Gastroesophageal Reflux Disease,

**BHR -** Bronchial hyper-reactivity,

**ACE-I –** ACE-Inhibitor drug.

The variability of the main causes of cough with age, gender and BMI was determined through correlation and presented in Table-III. Significant positive correlations were established between increasing age with ILD, use of ACE-Inhibitors and CCF, as well as younger age with cough variant asthma and allergic rhinitis. Significant correlation was also seen between female gender with both ILD and use of ACE-I, as well as male gender and Smoker’s cough. Higher BMI was significantly associated with ACE-I induced cough only but not with other causes of cough.

**Table-III:** Correlation between different causes of cough and patient characteristics.

Causes of Cough	Age in years r (p)	Gender r (p)	BMI r (p)
Cough variant asthma	-0.22** (0.00)	0.01(0.83)	0.02(0.75)
Allergic rhinitis	-0.14* (0.02)	0.06(0.32)	-0.13(0.05)
GERD	-0.06 (0.30)	0.09(0.15)	-0.00(0.95)
BHR	0.01 (0.88)	-0.00(0.93)	0.07(0.28)
ILD	0.36** (0.00)	-0.13* (0.04)	0.06(0.38)
ACE-I induced cough	0.15* (0.01)	-0.13*(0.04)	0.14*(0.04)
Tuberculosis	-0.00(0.92)	-0.02(0.68)	-0.09(0.18)
Smoker’s Cough	0.03(0.63)	0.18**(0.00)	-0.08(0.26)
Lung malignancy	0.04(0.51)	0.06(0.34)	-0.10(0.13)
Heart failure	0.14* (0.02)	-0.06(0.36)	0.00(0.93)

## DISCUSSION

In our study, cough variant asthma was the major cause of persistent cough, followed by allergic rhinitis, ILD, GERD, BHR and ACE-I induced cough. When variability of each cause of cough with age, gender and weight was determined, the results in our setup were almost similar to those of other setups with a few exceptions.

Cough variant asthma was the commonest cause of chronic cough in our study and had a significant relation with younger age group that was in keeping with the usual data about cough variant asthma suggesting that it is indeed more common in younger ages. CVA did not have a significant relationship with gender or BMI in this study even though it is hypothesized that a possible relationship exists between obesity and allergic diseases such as asthma.^6^ As our study cohort’s average BMI was in overweight rather than obese range and we had very few obese patients, it is likely the number of obese patients was too low to have found an association. Moreover, there is sound evidence that women’s association with asthma is more common than men and this may be confounded by greater age and obesity.^7^ Despite a female preponderance in our study group, we did not find this correlation to be significant. This could be again explained by the fact that we had few obese patients overall and as obese females are more at risk of developing asthma,^7^ we did not have significant number of obese patients to prove this correlation. Studies with larger study population are suggested to further evaluate these findings.

Allergic rhinitis, another common cause, had a significantly higher incidence in younger age group in our study which was comparable to Italian and American studies^8^,^9^ showing highest incidence in children and decreasing incidence in adults. Similar to previous studies,^8^,^9^ no significant gender predominance was found in our study. Literature yields conflicting results when ascertaining link between higher BMI and allergic rhinitis with some studies in favor of this association^10^ and some contradicting it.^11^ We did not find significant association between allergic rhinitis and BMI. We suggest further studies with larger study population to evaluate these findings.

Interstitial lung disease, another cause of cough in our study had a significant correlation with increasing age in our study, which is comparable to American population.^12^ In contrast to data from other populations,^12^ we found ILD more prevalent among females in our setup. This concurs with the local ILDPAK registry document.^13^ This suggests a trend in Pakistani population for ILD to be more prevalent in women although the reasons for this need to be further explored. We did not find any link between ILD and BMI; also literature search did not reveal any definitive data on this subject.

Gastroesophageal reflux disease associated cough had no significant relationship with age, gender or BMI in our study. No data has been found to establish the link between GERD associated cough and patient demographics on literature search, although GERD epidemiology is well defined. GERD has higher incidence in patients with increasing age^14^ and obese individuals^15^ whereas similar incidence amongst genders.^14^ Since GERD associated chronic cough is an important and often missed diagnosis, this highlights the importance of studying it further as data is quite lacking in defining patient demographics with this diagnosis.

Bronchial hyper-reactivity also had no significant relationship with age, Gender or BMI in our study although data suggest that advancing age is associated with increased bronchial hyper-responsiveness irrespective of coexisting pulmonary disease.^16^ Studies have showed conflicting results between association of BMI and bronchial hyper-reactivity; however, a major Italian study was carried out on a large scale that provided definitive evidence of association between BMI and BHR.^17^ As the overall number of patients with BHR in our study were few, a greater dataset with this diagnosis would be needed to establish differences based on patient demographics.

ACE-Inhibitor induced cough in our study was more frequent with increasing age in years, which is reasonably explained by the fact that indications of ACE-Inhibitors e.g. hypertension and heart disease are usually more common with increased age. We also found female gender and higher BMI to be at a greater risk of ACE-Inhibitor induced cough, which has been corroborated in a Swedish study due to effect of genetic tendency.^18^

Amongst other causes of cough, smokers’ cough was significantly associated with male gender. In Pakistan, smoking is more prevalent in males as compared to females,^19^ and our study suggested the same. We also found heart failure more prevalent in elderly, which is similar to worldwide data.^20^ None of the other causes of cough showed any significant patterns of occurrence based on age, gender or BMI.

## CONCLUSION

This study showed that cough variant asthma was the major cause of chronic persistent cough in our community. It also showed that the variability of epidemiology of cough variant asthma, allergic rhinitis ACE-inhibitor induced cough was mostly comparable to that of data available worldwide. There was a significant prevalence of interstitial lung disease in females contrary to other populations. Further research is needed in the field to delineate the local trends in this regard and compare to other population groups. It is important to identify causes of chronic cough by basic investigation like chest radiography and spirometry to proceed with appropriate management in order to reduce the burden of recurrent presentation to outpatients’ clinic.

### Authors’ Contribution:

**KT** conceived, designed, reviewed, did final approval of manuscript along with contribution to data collection and is responsible for integrity of research.

**AC** did data collection, statistical analysis and manuscript writing.
